# Vegetation Dynamics in Response to Climate Change and Human Activities in a Typical Alpine Region in the Tibetan Plateau

**DOI:** 10.3390/ijerph191912359

**Published:** 2022-09-28

**Authors:** Guosong Zhao, Lijie Ren, Zilong Ye

**Affiliations:** School of Geography and Information Engineering, China University of Geosciences, Wuhan 430074, China

**Keywords:** vegetation dynamics, residual analysis, Hurst exponent, human activities, ecological projects

## Abstract

Understanding past and future vegetation dynamics is important for assessing the effectiveness of ecological engineering, designing policies for adaptive ecological management, and improving the ecological environment. Here, inter-annual changes in vegetation dynamics during 2000–2020, contributions of climate change (CC) and human activities (HA) to vegetation dynamics, and sustainability of vegetation dynamics in the future were determined in Gannan Prefecture (a typical alpine region in the Tibetan Plateau), China. MODIS-based normalized difference vegetation index (NDVI), air temperature, precipitation, and land cover data were used, and trend analysis, multiple regression residuals analysis, and Hurst exponent analysis were employed. NDVI increased at a rate of 2.4 × 10^−3^∙a^−1^ during the growing season, and vegetation improved in most parts of the study area and some sporadically degraded areas also existed. The increasing rate was the highest in the Grain to Green Project (GTGP) areas. The vegetation in the southern and northern regions was mainly affected by CC and HA, respectively, with CC and HA contributions to vegetation change being 52.32% and 47.68%, respectively. The GTGP area (59.89%) was most evidently affected by HA. Moreover, a Hurst exponent analysis indicated that, in the future, the vegetation in Gannan Prefecture would continuously improve. The study can assist in formulating ecological protection and restoration projects and ensuring sustainable development.

## 1. Introduction

Vegetation is an important component of terrestrial ecosystems that influences terrestrial carbon and water balance and regulates climate [1,2,3]. Vegetation dynamics can indicate the terrestrial ecological environment status, which is in turn responsible for regional ecological security, especially in ecologically fragile areas [4,5]. Meteorological factors, such as air temperature, precipitation, and solar radiation, act as limiting factors for vegetation growth, thereby directly affecting the spatial distribution and dynamics of vegetation [6,7,8,9,10,11]. Moreover, human activities (HAs), such as excessive logging, population explosion, overgrazing, and mining, can negatively affect vegetation growth [12,13], whereas human interventions, such as land use management, can also positively influence vegetation growth, as observed in China and India [14]. For example, owing to the COVID-19 pandemic, the imposed restrictions resulted in a relatively early and greener spring in 60% of China in 2020 [15]. Ecological restoration projects promote vegetation growth [16,17,18]. As vegetation changes are affected by both climate change (CC) and HA, distinguishing the degree of influence of the two factors has become an important research topic to sustainably manage vegetation ecosystems under changing environments [2].

Geodetectors [19,20,21] and correlation analyses [17] are often used while studying these influences on vegetation. However, the Geodetector focuses on the attribution and characterization of the spatial distribution of vegetation changes while correlation analysis cannot quantify the individual contributions of the two factors to vegetation change. Therefore, to better measure the impact of CC and HA on vegetation changes, several quantitative analysis methods, such as threshold segmentation [22,23], biophysical model simulation [16,18,24,25], machine learning-based residual analysis [15,26,27,28,29,30], and linear regression-based residual analysis [31], have been proposed. Among these, linear regression-based residual analysis is the simplest and most effective method to indirectly estimate the impact of HA on vegetation change by simulating the impacts without considering CC and comparing the results with the actual observations [32]. This method has been applied in sub-Saharan West Africa [33], Syria [34], the Mongolian Plateau [8], northern and eastern China [4,22,35,36,37,38], the Loess Plateau [39], the Tibetan Plateau [40], and the Yangtze River Basin [41]. For example, Liu et al. [36] identified the degradation of eastern grasslands in China using time-series segmentation and residual trends. Using liner regression-based residual analysis, He et al. [40] revealed the influence of CC and HA on vegetation in the Three-River-Source area while Cao et al. [37] determined the individual contributions of HA and CC on vegetation greening in the the Beijing–Tianjin–Hebei region. Moreover, this method has been applied to distinguish the effects of CC and HA on water storage anomalies [42]. Most previous studies that analyzed and attributed vegetation changes to various sources have generally only focused on the past, and future trends in vegetation changes have not been considered. Notably, sustainable management of vegetation does require a complete representation of past and future regional vegetation changes. The Hurst exponent is an index that can be used to characterize the sustainability of future vegetation changes [1,7,17,43,44,45,46,47]. An analysis of the Hurst exponent combined with past trend analysis of vegetation changes can make vegetation dynamics clearer both in the past and the future, thereby benefitting future ecological protection and restoration project planning and ensuring sustainable development. For example, if an area exhibited vegetation improvement in the past 20 years, but these changes are not deemed sustainable (or persistent) according to a Hurst exponent analysis, then appropriate action cannot be taken in a meaningful way without an analysis of future trends.

Gannan Prefecture is located between the national Tibetan Plateau ecological barrier area and the key ecological zone of the Yellow River, and is important in conserving and replenishing the water source of the Yellow River, regulating climate, conserving soil and water, and maintaining biodiversity [20,48,49,50]. However, Gannan Prefecture has a fragile ecological environment and suffers from soil erosion. To mitigate ecological environment problems, enhance water conservation and replenishment capacity, and sustainably develop regional resources, environment, and population, several ecological protection and restoration projects have been conducted in this region [51,52]. Previous studies in Gannan Prefecture have majorly analyzed the spatial patterns of vegetation change and its drivers [20,53,54], or the changes in a specific ecosystem type, such as grasslands [55,56]. For example, Liu et al. [5] studied the spatial differentiation and dominant drivers of vegetation in Gannan Prefecture using spatial autocorrelation analysis and a Geodetector, and Li et al. [55] analyzed the growth status and drivers of grasslands in Gannan Prefecture and the Northwest Sichuan Region using partial correlation analysis and residual analysis. However, the degree of influence of both CC and HA on vegetation changes in Gannan Prefecture remains unclear; additionally, the conservation effectiveness of ecological restoration projects has not been assessed and the contribution of only HA to vegetation changes in ecological restoration projects has not yet been clearly explained. Furthermore, the future vegetation change trends have also not been analyzed. Therefore, the contribution of HA and CC to vegetation dynamics and future vegetation change trends should be assessed.

Considering Gannan Prefecture as the study area and combining meteorological data and remote sensing-based vegetation data, this study aimed to answer the following questions: (1) What are the change trends of vegetation growth from 2000 to 2020? What are the spatial patterns of vegetation changes? (2) What is the contribution of only HA to the dynamic vegetation changes during 2000–2020? (3) How will the vegetation growth in Gannan Prefecture change in the future?

The results of the study provide decision support and guiding suggestions to formulate future ecological restoration projects, promote adaptive management of vegetation, improve ecological environments, and ensure sustainable development.

## 2. Materials and Methods

### 2.1. Study Area

**Figure 1:**
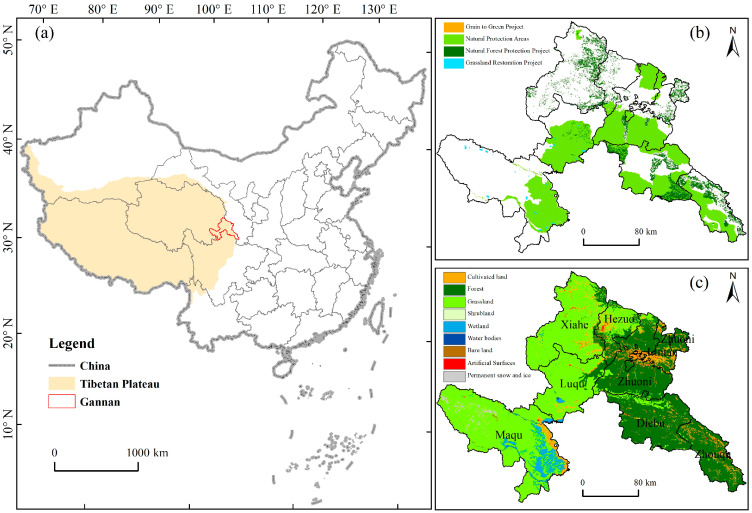
Location of Gannan Prefecture (**a**) and spatial distribution of various ecological protection and restoration projects (**b**), and land cover types (**c**).

Gannan Prefecture (100°76′–104°76′ E, 33°11′–35°59′ N), which is located in the northeast of the Tibetan Plateau, the upper reaches of the Yellow River, is a typical alpine region (average altitude of approximately 3000 m) [49,54]. The region shows a cold plateau continental climate. The annual sunshine hours are 2200–2400 h, average annual temperature is 1–3 °C, and average annual precipitation is 400–800 mm [56]. The major ecosystem types are grassland in the west and forest in the east, occupying more than 90% of the whole region. Several ecological protection and restoration projects, including the Grassland Restoration Project (GRP), Grain to Green Project (GTGP), Natural Forest Protection Project (NFPP), and Natural Protection Areas (NPA), were implemented to improve the vegetation status (Figure 1). The GRP include activities such as returning grazing to grasslands, black soil beach treatments, and fence construction. The GTGP converts arable land with serious soil erosion, sandy, saline, and rocky desertification, as well as arable land with low and unstable food production to forest or grassland in a planned and systematic manner according to local conditions. The NPA includes national or provincial forest parks, geoparks, and wetland protection areas. While in regions of NFPP, deforestation, conversion of natural forests into plantation forests, and other activities that damage natural forests and local ecological environment are prohibited.

### 2.2. Datasets

#### 2.2.1. Vegetation Remote Sensing Data

Remote sensing-based normalized difference vegetation index (NDVI) has been extensively used to reflect vegetation dynamics [57,58,59,60,61,62,63]. This study used the Google Earth Engine (GEE) platform to obtain NDVI data of Gannan Prefecture from 2000 to 2020 (https://doi.org/10.5067/MODIS/MOD13A1.006), at spatial and temporal resolutions of 500 m and 16 d, respectively. To accurately reflect the changing vegetation trends, the growth season was defined from April to October annually, and the average NDVI of the growing season was used to indicate the vegetation growth status [31]. Areas with NDVI < 0.1 in the growing season were excluded.

#### 2.2.2. Meteorological Data

Air temperature and precipitation data for 2000–2020 were acquired from the China Meteorological Data Service Center (http://data.cma.cn/ (accessed on 12 April 2021)) and interpolated using ANUSPLIN software to generate raster datasets of annual air temperature and precipitation with a spatial resolution of 500 m.

#### 2.2.3. Other Data

The land cover product launched in 2020 by GlobeLand30 was employed at a spatial resolution of 30 m to demonstrate the distribution of 10 land cover types (http://www.globallandcover.com/home_en.html (accessed on 21 March 2021)), namely, cultivated land, forest, grassland, shrubland, wetland, water bodies, tundra, artificial surface, bare land, permanent snow, and ice. Additionally, Landsat images in GEE were employed to study the vegetation changes.

### 2.3. Methods

#### 2.3.1. Trend Analysis

As mentioned in Section 1, linear regression has been widely used to study vegetation changes. In this study, linear regression was used to simulate the trends in vegetation change at the pixel scale, considering time and the average NDVI value as independent and dependent variables, respectively. The linear regression slope was used to represent the inter-annual trends in vegetation change. Positive and negative slopes indicated that the vegetation changes increased and decreased with time, respectively. The *slope* was calculated as follows:

(1) $$Slope=\frac{n\times \sum_{i=1}^{n}\left(i\times NDVI_{i}\right)-\sum_{i=1}^{n}i\sum_{i=1}^{n}NDVI_{i}}{n\times \sum_{i=1}^{n}i^{2}-\sum_{i=1}^{n}i}$$

where slope is the trend of the linear regression fitting equation of mean NDVI and time variables in the growing season, i is the time variable, with an integer value in the range of 1 − n, n is the number of years, and $NDVI_{i}$ is the average NDVI in the growing season in year i. NDVI trends were divided into five classes: notable degradation (*slope* < −0.005), slight degradation (−0.005 ≤ *slope* < −0.001), stable (−0.001 ≤ *slope* ≤ 0.001), slight improvement (0.001 < *slope* ≤ 0.005), and notable improvement (*slope* > 0.005).

#### 2.3.2. Residual Analysis

Residual analysis has been used to distinguish the effects of CC and HA on vegetation change. It assumes that vegetation growth depends on the climate and that after removing climate influences, vegetation changes are mainly caused by HA [32,64]. In this study, a binary regression model was established at the pixel scale, considering the average NDVI of the growing season as the dependent variable and the annual average temperature and annual precipitation as the independent variables. Based on the parameters fitted to the model, the predicted NDVI ($NDVI_{pre}$) was calculated to represent the effects of CC on vegetation change, while the residual NDVI ($NDVI_{res}$), which is the difference between the actual NDVI ($NDVI_{obs}$) and predicted NDVI $\left(NDVI_{pre}\right)$, represents the effect of HA on vegetation change. $NDVI_{pre}$ and $NDVI_{res}$ can be calculated as follows:

(2) $$NDVI_{pre}=a\times T+b\times P+c$$

(3) $$NDVI_{res}=NDVI_{obs}-NDVI_{pre}$$

where $NDVI_{obs}$ and $NDVI_{pre}$ represent the actual NDVI values acquired through remote sensing and model-based predicted NDVI values, respectively. Additionally, a, b, and c are the model fit parameters, and T and P represent air temperature and annual precipitation, respectively.

#### 2.3.3. Calculation of the Relative Contribution of Climate Change and Human Activities to Vegetation Change

The linear trend of $NDVI_{pre}$ and $NDVI_{res}$ can be calculated following Equation (1). By combining the linear trend with the trend of the actual NDVI observations, six conditions were obtained to quantitatively distinguish the relative contributions of CC and HA to vegetation changes in each pixel [65,66]. Table 1 lists the calculation methods.

#### 2.3.4. Hurst Exponent

The Hurst exponent (*H*), which can be used to represent the sustainability of vegetation changes, was first proposed by the British hydrologist Hurst to quantitatively describe the continuity of time series [67]. The sustainability of the NDVI time series is judged by the magnitude of *H* which is calculated using the R/S method [68,69].

The main calculation procedures are as follows:

a. Define a NDVI time series $NDVI_{t}\left(t=1,2,3,4,\ldots ,n\right)$ that divides the time series data into g-groups of r that do not overlap with each other ($NDVI_{i1},NDVI_{i2},\ldots ,NDVI_{ir}\left(i=1,2,\ldots ,g\right)$, $r=2,3,\ldots ,\frac{n}{2}$);

b. Define the mean sequence of subseries

(4) $$\bar{NDVI_{i}}=\frac{1}{r}\overset{r}{\underset{j=1}{\sum }}NDVI_{ij}\;\left(i=1,2,\ldots ,g\right)$$

c. Calculate the cumulative dispersion sequence

(5) $$X_{ij}=\overset{j}{\underset{k=1}{\sum }}\left(NDVI_{ij}-\bar{NDVI_{i}}\right)\;\left(i=1,2,\ldots ,g;j=1,2\ldots ,r\right)$$

d. Create the range sequence

(6) $$R_{i}=\max\left(X_{ij}\right)-\min\left(X_{ij}\right)\;\left(i=1,2,\ldots ,g;j=1,2\ldots ,r\right)$$

e. Calculate the standard difference sequence

(7) $$S_{i}={\left[\frac{1}{r}\overset{r}{\underset{j=1}{\sum }}{\left(NDVI_{iJ}-\bar{NDVI_{i}}\right)}^{2}\right]}^{\frac{1}{2}}$$

f. Calculate the Rescale range

(8) $$RS_{i}=R_{i}/S_{i}\;$$

(9) $$\bar{\mathrm{RS}}=\frac{1}{g}\sum_{i=1}^{g}RS_{i}$$

g. Calculate the Hurst exponent

(10) $$\bar{\mathrm{RS}}=cr^{H}$$

where *H* is the Hurst exponent obtained by fitting the equation $\left(\ln\bar{\mathrm{RS}}=a+H\times lnr\right)$ using the least-squares fitting method.

The *H* value includes three forms: (1) When 0.5 < *H* < 1, the time series is sustainable or persistent with the past long-term change; the greater the *H* value, the stronger the sustainability or persistence. (2) When *H* = 0.5, the time series is random and long-term correlation does not exist. (3) When 0 < *H* < 0.5, the time series has anti-sustainability or opposite direction with the past long time change; the smaller the *H* value, the stronger the anti-sustainability. In our study, *H* was divided into the following seven classes: strong anti-sustainability (0 < *H* ≤ 0.2), medium anti-sustainability (0.2 < *H* ≤ 0.35), weak anti-sustainability (0.35 < *H* < 0.5), random sequence (*H* = 0.5), weak sustainability (0.5 < *H* ≤ 0.65), medium sustainability (0.65 < *H* ≤ 0.8), and strong sustainability (0.8 < *H* < 1).

## 3. Results

### 3.1. Spatiotemporal Variations in Vegetation Dynamics

**Figure 2:**
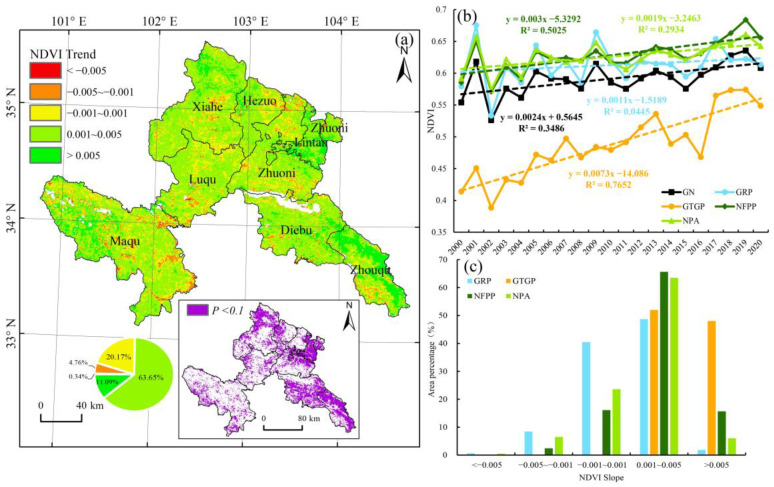
(**a**) Spatial distribution and significance test results of normalized difference vegetation index (NDVI) variation trends in Gannan Prefecture during 2000–2020. (**b**) Inter-annual changes in NDVI in different regions. (**c**) Proportion of different NDVI slopes in various ecological protection and restoration projects, including the Grassland Restoration Project (GRP), Grain to Green Project (GTGP), Natural Forest Protection Project (NFPP), and Natural Protection Areas (NPA).

The average NDVI during 2000–2020 in Gannan was 0.1–0.82, with an average value of 0.59. In the past 21 years, the average NDVI of the vegetation in the growing season fluctuated at a rate of 2.4 × 10^−3^∙a^−1^, with minimum and maximum values observed in 2002 and 2019, respectively; moreover, the vegetation recovered evidently during 2000–2020 (Figure 2b). Spatially, during this period, areas with an average NDVI slope > 0 accounted for 88.25%, showing vegetation greening in most parts of the study area (Figure 2a). Specifically, the vegetation areas showing slight or notable improvements accounted for 74.74%, and most of these areas passed the significance test at *P* < 0.1, indicating that the vegetation changes in most areas showed improvement. However, the NDVI values of a few sporadically distributed areas indicated slight or notable degradation. Such areas accounted for only 5.1%, while areas with stable vegetation accounted for 20.71%.

During 2000–2020, vegetation under all ecological protection and restoration project areas showed improvements, and the inter-annual change rate of NDVI differed marginally across different ecological restoration projects, with the largest change rate observed in the GTGP (Figure 2b). Spatially, most areas in these projects showed increasing NDVI trends and extremely few areas showed a declining trend, and the change trend of the GTGP was the most evident. According to the statistical results, the NDVI change trend in each ecological restoration project was mostly concentrated in the slight improvement category (0.001 < *slope* ≤ 0.005), indicating improvements in the vegetation condition and conservation effectiveness of the ecological protection and restoration projects (Figure 2c).

### 3.2. Relative Contributions of Climate Change and Human Activities to Vegetation Dynamics

**Figure 3:**
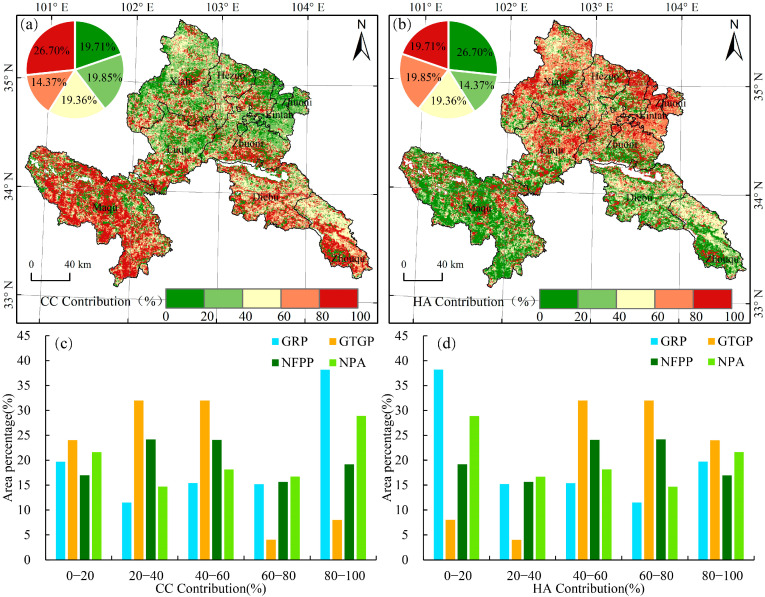
Spatial distribution and statistics of the relative contribution of climate change (CC) (**a**,**c**) and human activities (HA) (**b**,**d**) on vegetation changes under different projects during 2000–2020.

CC and HA together drove the vegetation changes. Overall, both factors improved the vegetation in the study area. The average contribution of CC to the vegetation changes was 52.32%, with the regions with high contribution distributed mainly in the southern regions (60–100%) (Figure 3a). The average contribution of HA to the vegetation changes was 47.68%, with areas with high contribution distributed mainly in the northern regions (Figure 3b). Moreover, in areas with NDVI slope > 0, CC mainly drives the vegetation improvement with a contribution of 56.57%, implicating CC is the main contributor in vegetation improvement compared with human activities. However, in areas with NDVI slope < 0, HA is the main the contributor in vegetation degradation, occupying 79.65%, much larger than the CC contribution.

For the past 21 years, HAs, such as ecological protection and restoration projects, have actively improved the vegetation in Gannan Prefecture. The degree of vegetation changes influenced by HA varies across different projects, with vegetation changes being most affected by the GTGP (59.89%). The contribution of HA to vegetation changes in the GTGP was concentrated in ranges of >40%, accounting for 88% of the total study area, whereas that of the NFPP was concentrated in the range of 60–100%, mainly in Xiahe, Luqu, Zhuoni, and Lintan counties. In the GRP, the degraded grassland and black soil beach in the Bohai Village of Langmusi Town, the black soil beach governance in Nima Town, and the poisonous weed plant governance area in the Gahai Township were greatly affected by HA, while in the remaining project areas, HA was mostly concentrated in the range of 0–40%. In the nature reserves, represented by Gahai-Zecha National Nature Reserve and the northern part of Taohe National Nature Reserve, the vegetation change was relatively more influenced by HA, in a range of 80–100%.

### 3.3. Future Sustainability Analysis of Vegetation Dynamics

**Figure 4:**
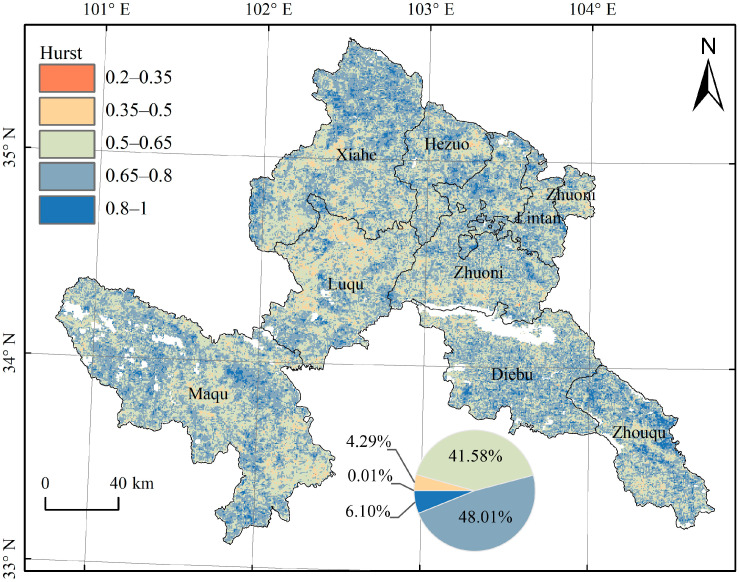
Spatial distribution and statistics of the Hurst index according to NDVI time series.

The *H* value was calculated at the pixel scale based on the NDVI time series data of 2000–2020. The spatial distribution of the calculated *H* value is shown in Figure 4. The maximum and minimum values were 0.97 and 0.26, respectively, with an average value of 0.66, and a value >0.5 accounting for 95.7%. Since the index value throughout the study area was >0.2, strong anti-sustainability and random sequence (H = 0.5) did not exist in this study. Further, as the average value was >0.5, the vegetation changes in Gannan Prefecture and various ecological restoration project areas were mainly sustainable, indicating that future vegetation changes will improve continuously as observed in the past 21 years.

**Figure 5:**
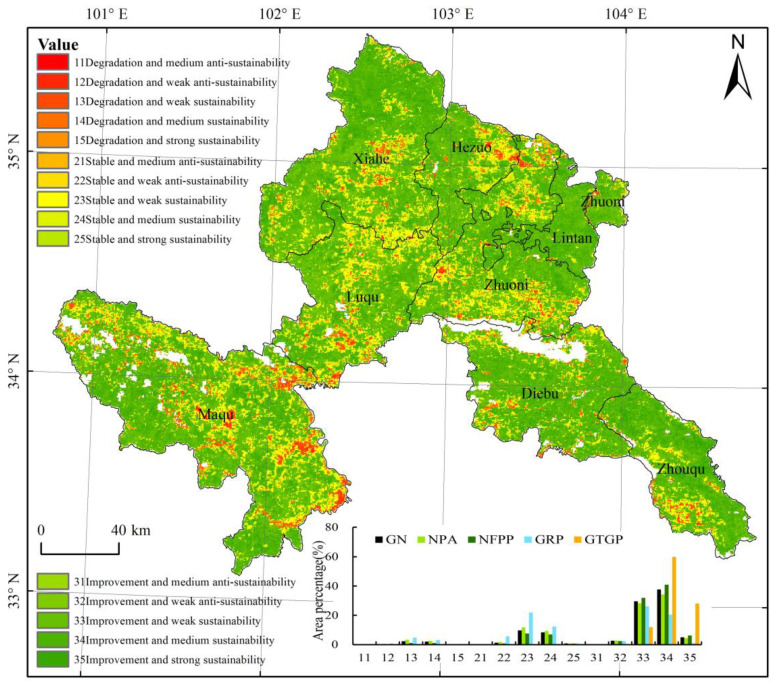
Spatial pattern of the 15 cases acquired by combining the past vegetation change trends and future sustainability of vegetation changes.

To further reveal the past vegetation change trends and future sustainability of vegetation changes, we overlaid these two factors to reflect the past and future vegetation changes together. The change trend of NDVI during 2000–2020 was divided into three categories: degradation (<−0.001), stable (−0.001–0.001), and improvement (>0.001). On combining these with the sustainability types as indicated by the *H* value, 15 cases were acquired (Figure 5). The results showed that vegetation changes in Gannan Prefecture were dominated by the following three categories (72.18%): improvement and weak sustainability (29.52%), improvement and medium sustainability (37.62%), and improvement and strong sustainability (5.04%).

The vegetation changes in each ecological protection and restoration project area mainly showed continuous improvement. However, a few cases of vegetation degradation with sustainability and vegetation improvement with anti-sustainability, accounting for 5% of the total ecological restoration project area, require further attention. These areas were sporadically distributed in a small part of the Indigenous Fish Nature Reserve of the Tibetan Plateau in Maqu County; Zhiliguan National Forest Park in the nature reserve project and fence construction in Shangnairima Village, Cairima Township; black soil beach management in Nima Township; and Xiuma Village in the GRP area.

## 4. Discussion

### 4.1. Impact of Human Activities on the Vegetation Dynamics

Similar to the Gannan studies of Liu et al. [5], Wang et al. [53], Liang and Wang [54], this study also reported an NDVI increase after 2000. Moreover, the vegetation degradation in the southwestern part is also found in a study of Liu et al. [5] and Wang et al. [53]. CC and HA together drove the vegetation change. Specifically, the relative contribution of HA to vegetation change showed a north–south spatial pattern, with the northern and southern regions being relatively more and less affected by HA, respectively. As shown in Figure 1, the cultivated land, a highly human impacted land type, was mainly distributed in the northern regions, including Lintan, Zhuoni, and Xiahe counties, and a small proportion of cultivated land was distributed in the eastern part of Maqu and Zhouqu counties. According to Figure 3, areas where HA contributed more to vegetation changes were mainly distributed in areas showing intensive human factors, such as farmlands and artificial surfaces. Studies by Jin et al. [31], Yi et al. [41], Li et al. [62], Liu et al. [70], and Qu et al. [71] also showed a similar rule that HA is the main contributor to vegetation change in agricultural areas or urbanization areas. Thus, the findings proved that the residual analysis method using a regression between NDVI and meteorological data, and the use of residuals to indirectly estimate the impact of HA on vegetation changes were suitable.

**Figure 6:**
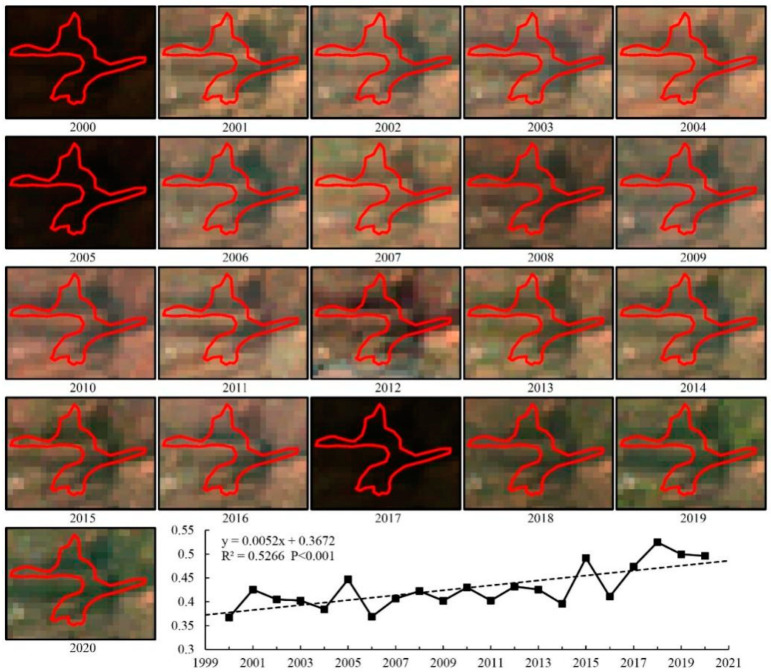
Landsat RGB composite images and inter-annual variations in vegetation MODIS NDVI in a project area of cultivated land returning to forest during 2000–2020.

This study also found a positive role for ecological protection and restoration projects to promote vegetation growth [16,17,18,39,72]. Among the various ecological restoration projects in Gannan Prefecture, the GTGP was the most affected by HAs (59.89%), which are also found in the areas of Loess Plateau [57,73] and Karst regions [74,75]. To exhibit the vegetation changes in the GTGP more clearly, Landsat 5/7/8 remote sensing images of the growing season for 2000–2020 were selected based on the GEE platform, and true color synthesis was applied to reflect the land surface characteristics and real-time changes in vegetation to ascertain the protection effect of the GTGP. In this study, one of the project areas was analyzed. Figure 6 shows that the inter-annual variations in vegetation NDVI showed major improvement in this project at improvement rates of 5.2 × 10^−3^∙a^−1^, thus showing a significant increasing trend (*P* < 0.001). According to the remote sensing data of the past 21 years, the vegetation of the project changed from brown to green. Thus, the positive effects of the GTGP were evident, indicating that HA directly affected vegetation changes (Figure 6). In areas implementing NFPP, mountains are closed off for afforestation, and indiscriminate cutting and illegal logging activities are forbidden, these efforts facilitate the forest’s significant recovery. For NPA, human activities such as built-up land expansion, cropland reclamation, and forest logging are not allowed, which is beneficial for promoting vegetation improvement (Figure 2). Compared with the GTGP, NFPP, and NPA, the implementation effect of GRP is lower, which may be related to the increasing stock capacity and overgrazing [53,54]. In GRP areas with an NDVI Slope < 0, HA contribution is approximately 73%, indicating the importance of the balance between the grassland forage supply and livestock-carrying pressure for future grassland management.

### 4.2. Impact of Climate Change on the Vegetation Dynamics

**Figure 7:**
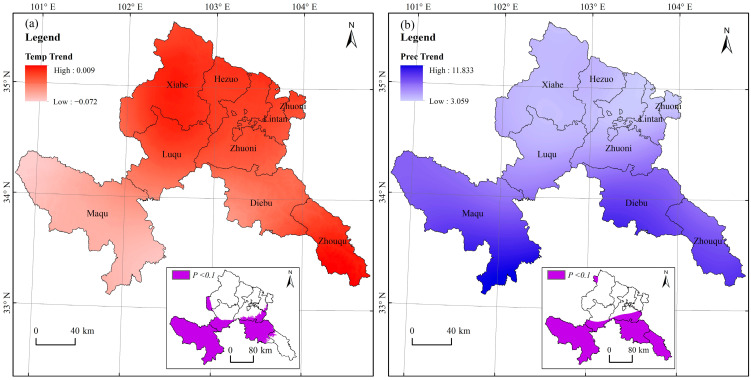
Change trend of air temperature (**a**) and precipitation (**b**) in Gannan Prefecture during 2000–2020.

CC also plays an important role in driving vegetation change [6,9,22,24,35,37,38,41,71,76]. Similar to the relative contribution of HA, the relative contribution of CC to vegetation change also showed a clear north–south spatial pattern; in contrast, the southern and northern regions were relatively more and less affected by CC, respectively. Figure 1 shows that the southern regions were dominated by grasslands, woodlands, and wetlands. As shown in Figure 7, Gannan showed a cooling and wetting trend over the past 21 years, which is consistent with the findings of Chen et al. [45]. The spatial pattern of the significance test on change trend of precipitation and air temperature also showed a clear significant north–south pattern, with the southern part passing the significance test of *P* < 0.1 (Figure 7). This fact indicated that the vegetation changes in the southern regions may be more affected by significant air temperature and precipitation change rather than HA. Moreover, areas in the southwestern and southeastern part experienced similar significant precipitation increase, but the NDVI change amplitude in the southeastern part is significantly higher than that in the southwestern part, and the same was the case for areas with a significance test of *P* < 0.1 (Figure 2). This may be due to different air temperature change amplitudes in these two regions. Large decrease in air temperature in the southwestern part prohibited the vegetation growth, thereby offsetting the positive role of more available water for vegetation growth from increasing precipitation [6,31,45,77].

Among the ecological protection and restoration projects, the contribution of CC to vegetation changes in NPA and GRP was greater than that of HA. Further, the contribution of CC to vegetation improvement in the GRP area was approximately 60%, which could be related to the implementation year of GRPs in Luqu and Maqu counties in this study or the poor ability to execute the project in some places. Most of these project areas in Luqu and Maqu were implemented in 2017; therefore, the inter-annual variations of the GRP fluctuated considerably before 2017, and increased after 2017 (Figure 2b). In addition, according to the contribution statistics in GRP, CC contributed only 27% in areas with NDVI slope < 0, that is because the serious overloading of livestock in some places make the grassland ecological pressure still very high, and it is difficult to reverse the local vegetation deterioration within a certain period of time [54].

### 4.3. Limitations and Implications

In this study, considering that air temperature and water playing an important role in vegetation growth, we used these two meteorological factors as independent variables in a multiple linear regression with NDVI to simulate vegetation changes under the influence of climatic factors. Then the effects of anthropogenic factors were disaggregated based on residual analysis. However, other factors also influence vegetation growth [10,38,78,79], such as solar radiation or sunlight hours, soil moisture, etc. For example, previous studies [80,81] have reported that drought-induced soil water deficit is often a main factor affecting vegetation growth. The influence of other factors on vegetation change should be considered in future studies. In addition, the multiple linear regression residual analysis method assumes a linear relationship between NDVI and climate variables which ignores the complex and nonlinear interactions; the machine learning method offers the opportunity to establish a nonlinear relationship between vegetation condition (i.e., NDVI) and influence factors [27,82], which deserves in-depth study.

**Figure 8:**
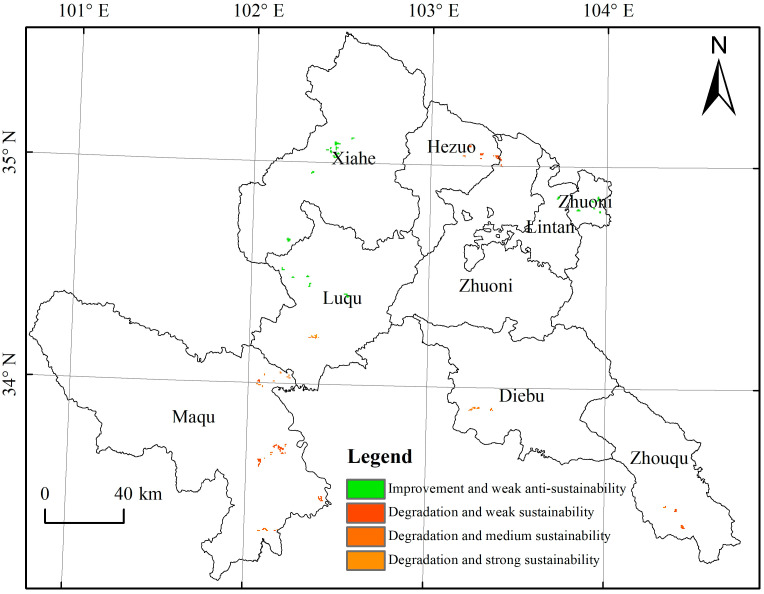
Areas with present degradation and future sustainability trends during 2000–2020.

The integrated method of trend analysis combined with the Hurst exponent is a valuable and reliable way for future land use planning and management practices [45,46,47]. Based on past vegetation changes, the sustainability of future vegetation changes was also analyzed in this study. By combining both these datasets, areas that showed conditions of present significant degradation (*P* < 0.1) and future sustainability, or present significant improvement and future anti-sustainability were extracted. Figure 8 shows that sustainable degradation vegetation changes in the future were mainly distributed in Maqu, Luqu, Diebu, Zhouqu, and Hezuo counties. Weak, medium, and strong sustainability areas accounted for 59.26%, 34.72%, and 6.02% of the total targeted areas, respectively. In the future, the above areas should be given prior attention for protection and restoration. Furthermore, unsustainable improvement vegetation changes are distributed in Xiahe and Luqu counties, needing extra protection (Figure 8).

## 5. Conclusions

Based on the air temperature, precipitation, and NDVI data, this study analyzed the vegetation changes during 2000–2020 and their sustainability in the future in Gannan Prefecture and ecological protection and restoration project areas, using trend analyses, residual analyses, and the Hurst exponent. The following conclusions were obtained:

(1) The overall vegetation growth improved majorly, with few degraded areas being scattered. The average NDVI in the growing season increased at a rate of 2.4 × 10^−3^·a^−1^. The NDVI in each ecological restoration project area increased, thus indicating the effectiveness of various ecological projects. In particular, the protection effect of the GTGP was the most evident.

(2) In the past 21 years, the relative contributions of CC and HA to the vegetation changes have differed spatially. The effects of CC and HA on vegetation change showed distinct spatial patterns, with the southern and northern regions mainly affected by CC and HA, respectively; correspondingly, the contributions to vegetation change were 52.32% and 47.68%, respectively. Among the ecological restoration projects, the GTGP was most evidently affected by HA (59.89%).

(3) According to the Hurst analysis, the vegetation changes in Gannan Prefecture and various ecological project areas will continue to improve. In the future, more attention should be paid to the areas presently showing degradation and having a scope for future sustainability, or areas presently showing improvement and having a scope for future anti-sustainability.

## Figures and Tables

**Table 1:** Relative contributions of climate change and human activities to changes in vegetation NDVI.

$slope\left(NDVI_{obs}\right)$	$slope\left(NDVI_{pre}\right)$	$slope\left(NDVI_{res}\right)$	Contribution Rate of Climate Change (%)	Contribution Rate of Human Activities (%)
>0	>0	>0	$\frac{slope\left(NDVI_{pre}\right)}{slope\left(NDVI_{obs}\right)}$	$\frac{slope\left(NDVI_{res}\right)}{slope\left(NDVI_{obs}\right)}$
	>0	<0	100	0
	<0	>0	0	100
<0	<0	<0	$\frac{slope\left(NDVI_{pre}\right)}{slope\left(NDVI_{obs}\right)}$	$\frac{slope\left(NDVI_{res}\right)}{slope\left(NDVI_{obs}\right)}$
	<0	>0	100	0
	>0	<0	0	100
